# Supplementary material for: The ASC inflammasome adapter governs SAA-derived protein aggregation in inflammatory amyloidosis
Source: EMBO Mol Med. 2024 Jul 30;16(9):2024–42. doi: 10.1038/s44321-024-00107-0 (PMC11393341; doi:10.1038/s44321-024-00107-0)
Supplement: Supplementary file 1 — Appendix [file 44321_2024_107_MOESM1_ESM.pdf]

Appendix

The ASC inflammasome adapter governs SAA-derived protein aggregation in inflammatory amyloidosis

Marco Losa[1], Marc Emmenegger[1], Pierre De Rossi[2,#], Patrick M Schürch[3,#], Tetiana Serdiuk[4,#], Niccolò Pengo[5], Danaëlle Capron[5], Dimitri Bieli[5], Niklas Bargenda[2], Niels J Rupp[6,7], Manfredi C Carta[1], Karl J Frontzek[1], Veronika Lysenko[3], Regina R Reimann[1], Petra Schwarz[1], Mario Nuvolone[1,8], Gunilla T Westermark[9], K. Peter R. Nilsson[10], Magdalini Polymenidou[2], Alexandre P. A. Theocharides[3], Simone Hornemann[1], Paola Picotti[4], and Adriano Aguzzi[1,†]

[1] Institute of Neuropathology, University Hospital Zurich, Zurich, Switzerland

[2] Department of Quantitative Biomedicine, University of Zürich, Zurich, Switzerland

[3] Department of Medical Oncology and Hematology, University Hospital Zurich, Zurich, Switzerland

[4] Institute of Molecular Systems Biology, Department of Biology, ETH Zurich, Zurich, Switzerland

[5] Mabylon AG, Schlieren, Zurich, Switzerland

[6] Department of Pathology and Molecular Pathology, University Hospital Zurich, Zurich, Switzerland

[7] Faculty of Medicine, University of Zurich, Zurich, Switzerland

[8] Amyloidosis Research and Treatment Center, Fondazione Instituto di Ricovero e Cura a Carattere Scientifico (IRCCS) Policlinico San Matteo, University of Pavia, Pavia, Italy

[9] Department of Medical Cell Biology, Uppsala University, Uppsala, Sweden

[10] Department of Physics, Chemistry and Biology, Linköping University, Linköping, Sweden

[#] Equal contribution

[†]Corresponding author: Adriano Aguzzi, Institute of Neuropathology, University of Zurich

Schmelzbergstrasse 12, CH-8091 Zurich, Switzerland, E-mail: adriano.aguzzi@usz.ch

Table of contents

Page	Content
**1**	Title page
**2**	Table of contents
**3**	Appendix Fig S1
**4**	Appendix Fig S2
**5**	Appendix Fig S3
**6**	Appendix Fig S4
**7**	Appendix Fig S5
**8**	Appendix Fig S6
**9**	Appendix Fig S7
**10**	Appendix Fig S8
**11**	Appendix Fig S9
**12**	Appendix Fig S10
**13**	Appendix Fig S11
**14**	Appendix Fig S12
**15**	Appendix Fig S13
**16**	Appendix Fig S14
**17**	Appendix Table S1
**17**	Appendix Table S2
**17**	Appendix Table S3
**18**	Appendix Table S4
**18**	Appendix Table S5
**19**	Appendix Table S6
**19**	Appendix Table S7
**19**	Appendix Table S8
**20**	Appendix Table S9
**20**	Appendix Table S10
**21**	Appendix Table S11
**22**	Appendix Table S12

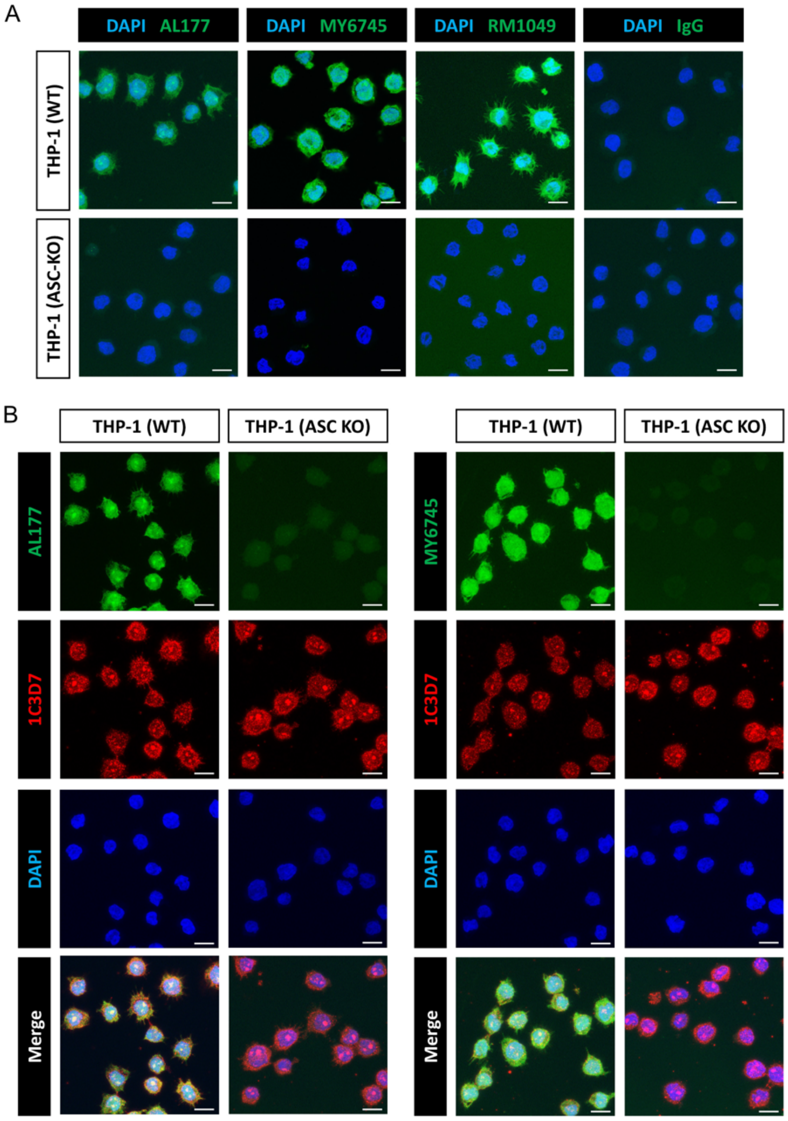

Appendix Fig S1. Specificity assessment of anti-ASC antibodies. (A) The three anti-ASC antibodies (green; AL177, MY6745 and RM1049) specifically identified ASC in THP-1 cells that highly express the inflammasome adaptor ASC. Nuclear staining with DAPI (blue). IgG isotype control is shown. **(B)** AL177 (left panels) and MY6745 (right panels) show specific anti-ASC recognition, whereas 1C3D7 anti-ASC antibody (omitted in our study) did display unspecific binding in *ASC*$^{-/-}$ THP-1 cells. Scale bar: 10 µm.

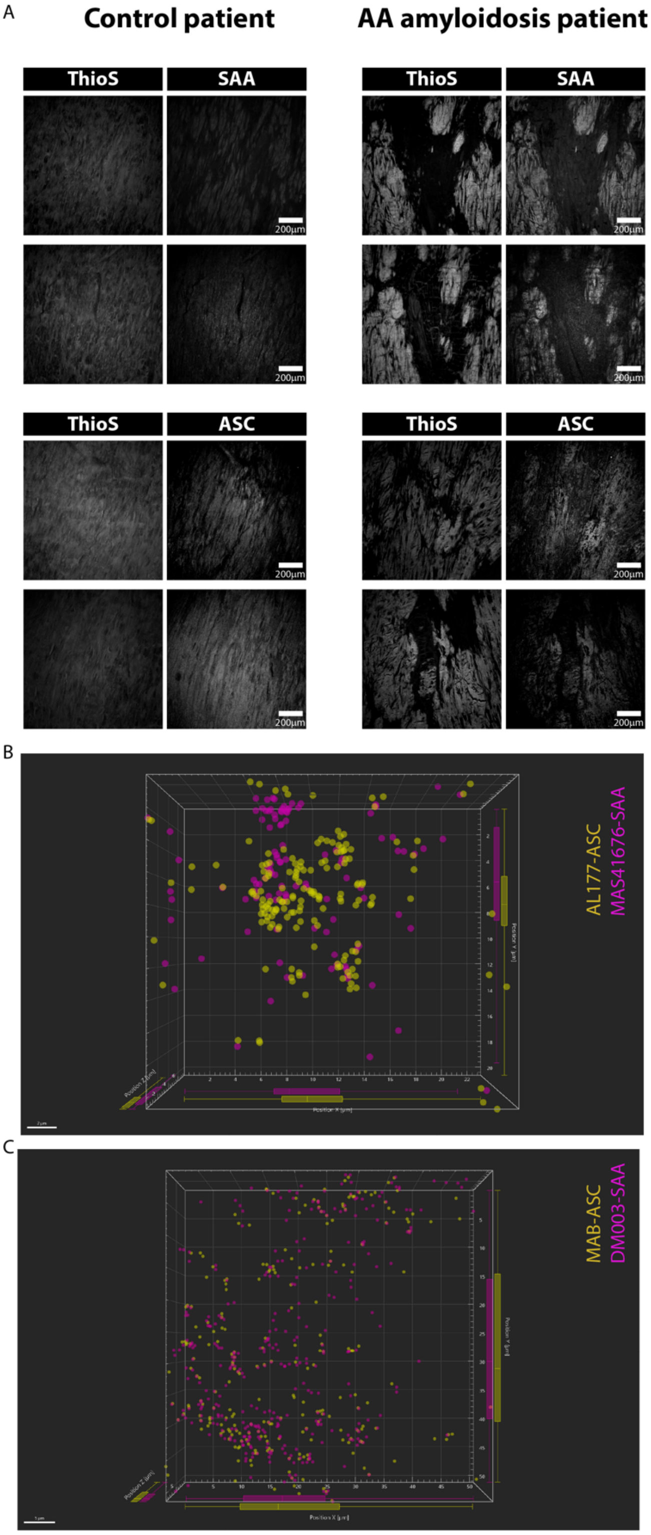

Appendix Fig S2. Colocalization of Thioflavin S staining with ASC and SAA and 3D representation of ASC and SAA staining. (A) Confocal images of patient with or without AA amyloidosis showing Thioflavin S staining, revealing protein aggregates forming β-sheets, and SAA or ASC. All grey levels were adjusted identically for both subjects. **(B)** and **(C)** Plot presenting 3D geolocalisation of center of mass of the dots reconstructed on the STED signal for ASC and SAA. On the side, boxplots represent the distribution in X, Y, and Z direction. The distributions of ASC and SAA are highly congruent.

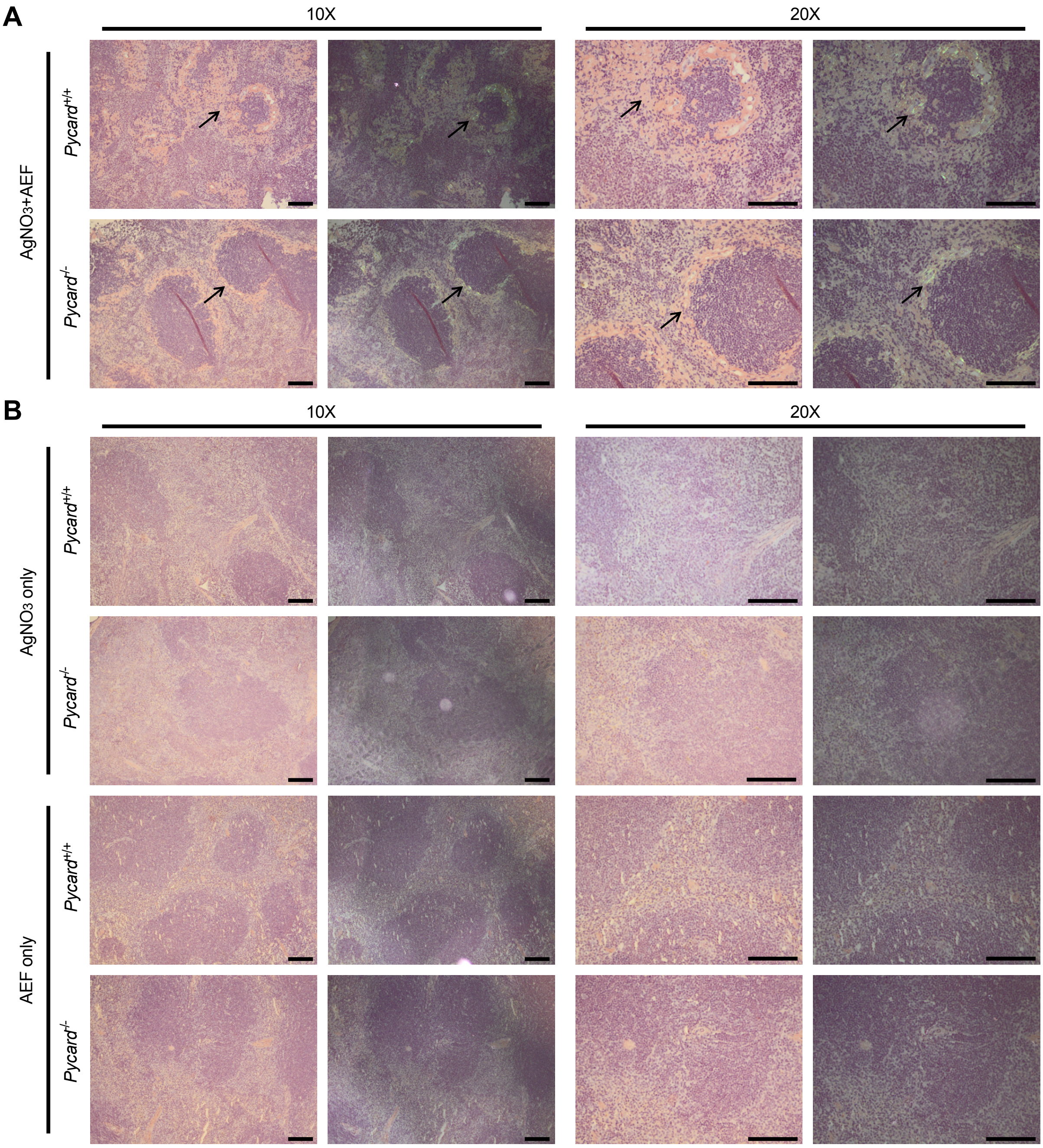

Appendix Fig S3. **Amyloid stained by Congo red shows apple-green birefringence in polarized light only in mice with AA induction**. **(A)** Representative Congo red-stained photo micrographs from indicated experimental groups showing amyloid apple-green birefringence (black arrows) under polarized light in AA^+ mice that received both, AEF and $AgNO_3$ injections (Jagusiak *et al*, 2019). In light microscopy, amyloid manifests as amorphous pink material (black arrows). **(B)** Important to note, there is no amyloid in the $AgNO_3$ only nor in the AEF only treated group (AA^- mice). Scale bar 100 µm.

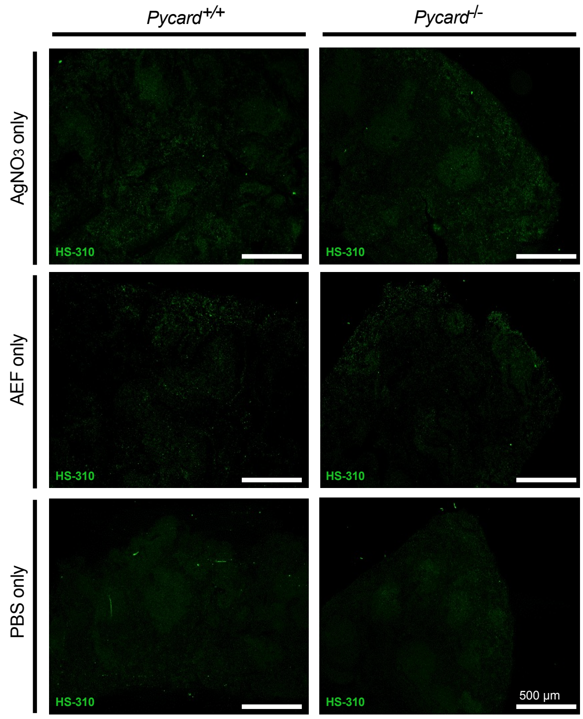

Appendix Fig S4. **Absence of amyloid deposition in mice only treated with $AgNO_3$, AEF or PBS (AA$^-$ mice)**. Visualization of amyloid deposition by hexameric LCP HS-310. No amyloid was seen in the control groups ($AgNO_3$-only, AEF-only and PBS-only). Light exposure time and 4x microscope objective were kept equal throughout the imaging/experiment.

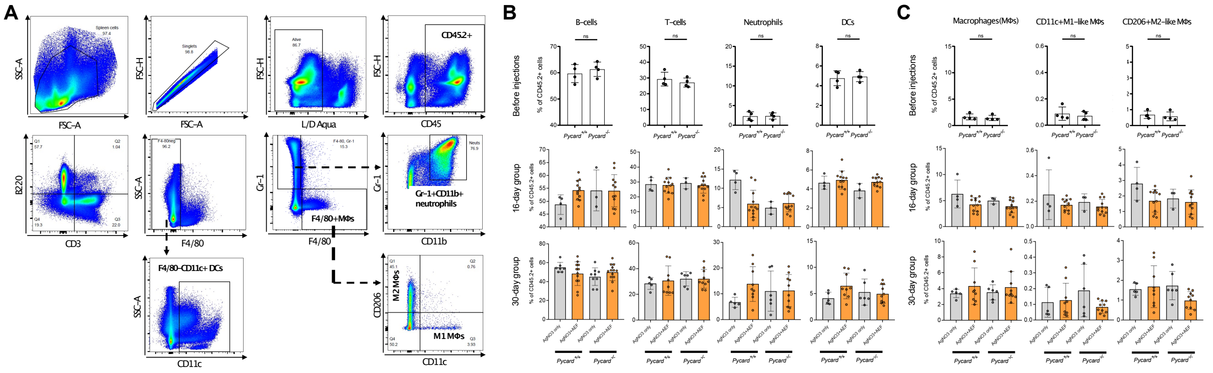

Appendix Fig S5. **Assessment of splenic cellular architecture before injections and AA disease state**. **(A)** Representative flow cytometry analysis and gating strategy of murine spleen cells. CD45.2 is an alloantigen expressed on all hematopoietic cells (except mature erythrocytes and platelets). B cells defined as CD45+CD3-B220+, T cells defined as CD45+B220-CD3+, Dendritic cells (DCs) defined as CD45+F4/80-CD11c+ and Neutrophils defined as CD45+Gr-1+CD11b+. Macrophages (MΦs) defined as CD45+F4/80+, and M1-like MΦs defined as CD45+F4/80+CD206-CD11c+ whereas M2-like MΦs defined as CD45+F4/80+CD11c-CD206+. **(B)** Relative abundance of B cells, T cells, neutrophils and dendritic cells (DCs) are plotted as percentage of CD45.2 positive events. Three different time points are represented. First row of panels: Before injections. Second row of panels: 16-days group. Third row of panels: 30-days group. There is no statistically significant difference in splenic cellular composition in unpaired two-tailed Student's t-test between the equivalent experimental group of *Pycard*$^{+/+}$ and *Pycard*$^{-/-}$ mice at each individual time point. Error bar represents standard error of the mean (SEM). Each dot represents an individual mouse. **(C)** F4/80+ macrophages, M1-like MΦs and M2-like MΦs are plotted as percentage of CD45.2 positive events at three different time points. First lane panel represents baseline values whereas data of animals euthanized after 16 and 30 days are plotted in the second and third lane panels, respectively. Results are represented as mean ± standard error. Each dot represents one individual mouse.

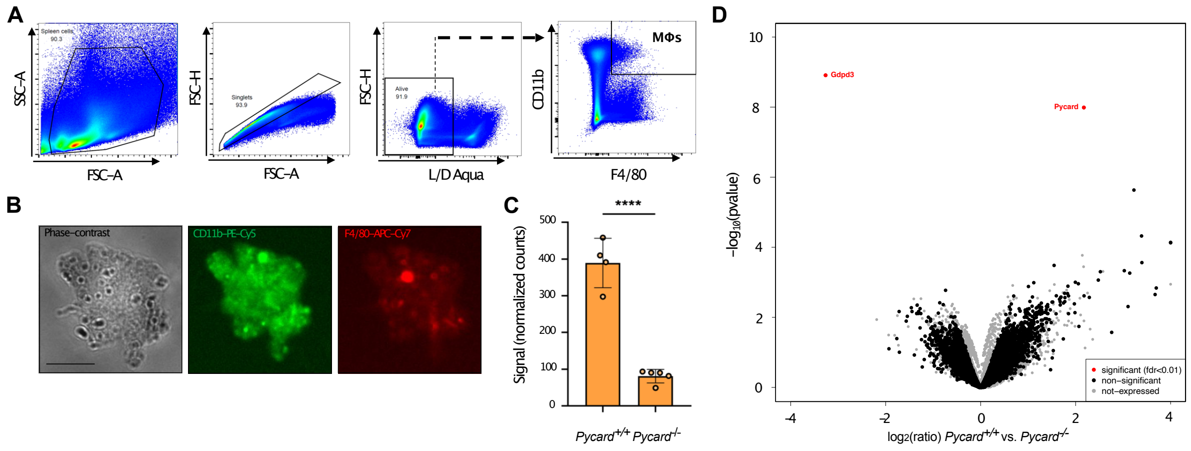

Appendix Fig S6. Transcriptional analysis of splenic macrophages from AA^{+} mice reveals mainly changes in *Pycard* expression. (A) Representative gating strategy of splenic macrophages flow cytometrically sorted from AA^{+} mice. Splenic cells were first gated in an FSC-A vs. SSC-A plot followed by doublet and dead cell exclusion. Finally, CD11b+F4/80+ macrophages were bulk sorted for transcriptomic analysis. **(B)** Representative images of splenic macrophages assessed **by** phase contrast and fluorescence microscopy. . The middle and right panel confirms the presence of the two antibody-conjugated fluorophores PE-Cy5 and APC-Cy7 that were used to identify CD11b+ and F4/80+ macrophages, respectively. Scale bar: approximately 10 µm. **(C)** Scatter plot depicting normalized counts of ASC transcript reads. An unpaired, two-tailed Students' t-tests was performed. Each dot represents one individual mouse. **(D)** **'**Volcano plot' of statistical significance vs. foldchange between *Pycard*$^{+/+}$and *Pycard*$^{-/-}$ splenic macrophages from AA^{+} mice displays the most significantly and differentially expressed genes (in red). * *P*<0.05, **** *P*<0.0001, ns: not significant.

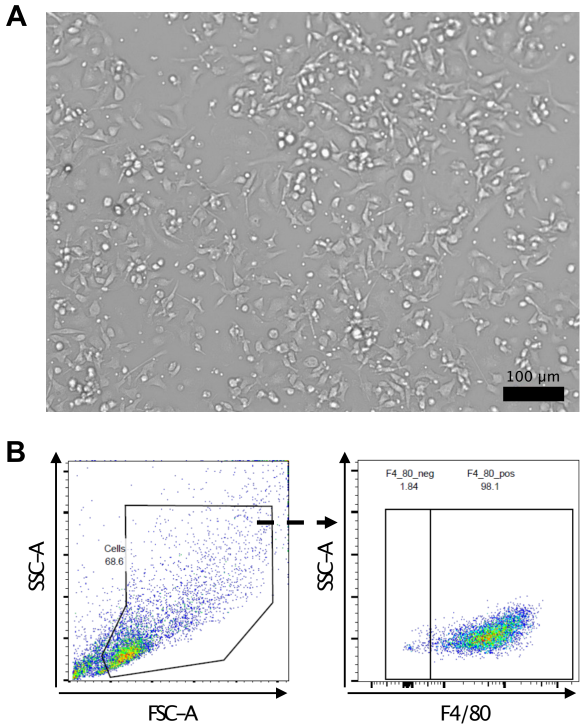

Appendix Fig S7. ***In vitro* phagocytosis of SAA-stimulated BMDMs**. **(A)** Phase contrast micrograph of adherent and differentiated BMDMs. **(B) Representative** flow cytometry gating for BMDM differentiation performed with anti-mouse F4/80 antibody, a specific macrophage marker. Alive F4/80+ cells represent differentiated BMDMs.

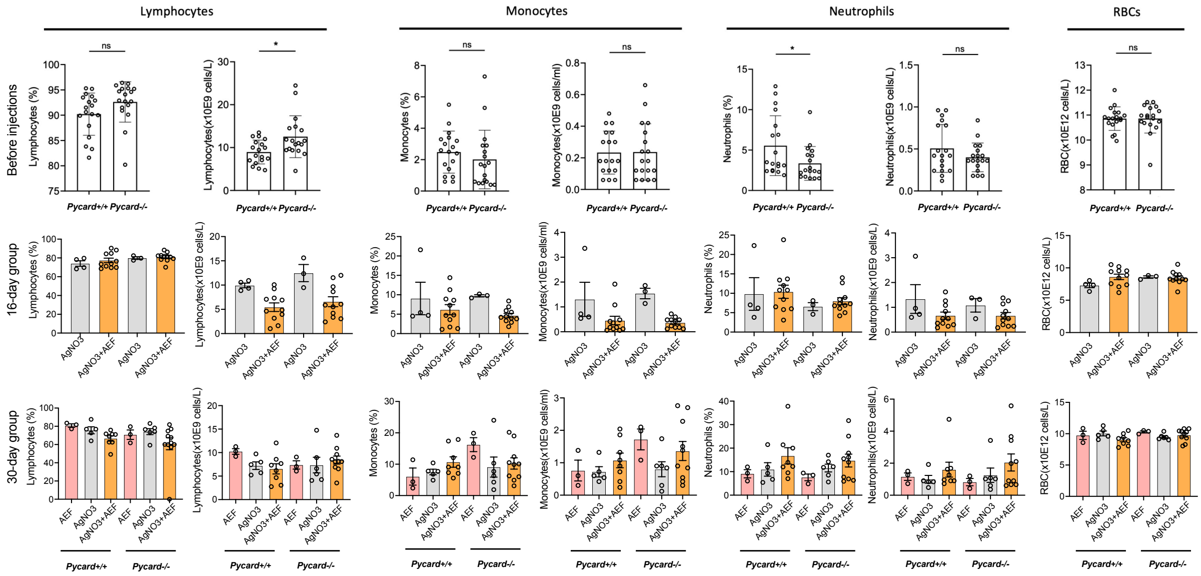

Appendix Fig S8. **Complete blood count (CBC) assessment did not reveal significant changes in cellular blood compartments between AA-diseased and $AgNO_3$-only control animals**. Bar plots depicting the abundance of lymphocytes, monocytes, neutrophils red blood cells (RBCs) plotted in relative (%) as well as absolute values (cell numbers). Top panel represents before injections values of *Pycard*$^{+/+}$and *Pycard*$^{-/-}$ mice. Data of mice euthanized at day 16 and 30 are plotted in the middle and bottom panels, respectively. Results are represented as mean± standard deviation and SEM (error bars). Statistical analysis performed by unpaired two-tailed Student's t-test in CBC between the equivalent experimental group. Each dot represents one individual mouse. * P<0.05, ns: not significant.

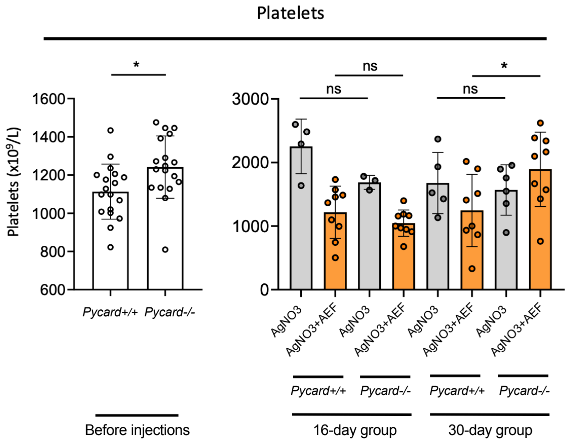

Appendix Fig S9. **Altered platelet count in the absence of *Pycard* before injections and in AA disease state**. Bar plots depicting platelet counts from experimental mice and different time points are represented. Results are represented as mean± standard deviation (error bars). Platelet counts < 150 $x10^9$/L were excluded from analysis. Analysis was performed using unpaired two-tailed Student's t-tests. Each dot represents one individual mouse. * P<0.05, ns: not significant.

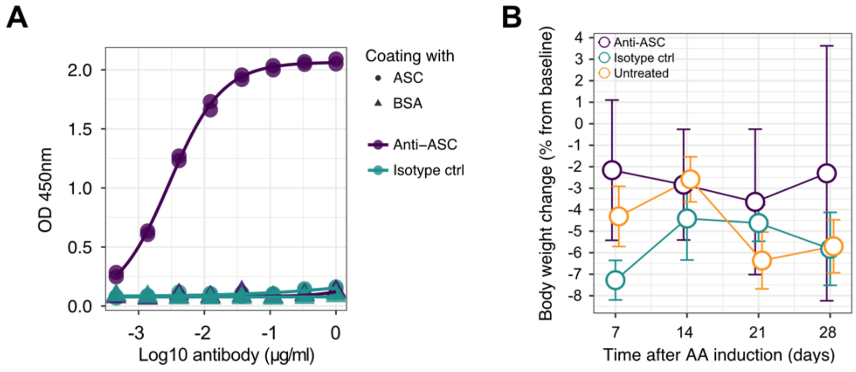

Appendix Fig S10. **Anti-ASC antibody binding curve and body weight changes during immunotherapy. (A)** ASC-specific binding assay for custom ASC and isotope control antibody. **(B)** Body weight change during the experiment (± SEM). Groups of 3 animals were treated with anti-ASC antibodies, isotype control, or were left untreated. Statistics: Kruskal-Wallis test.

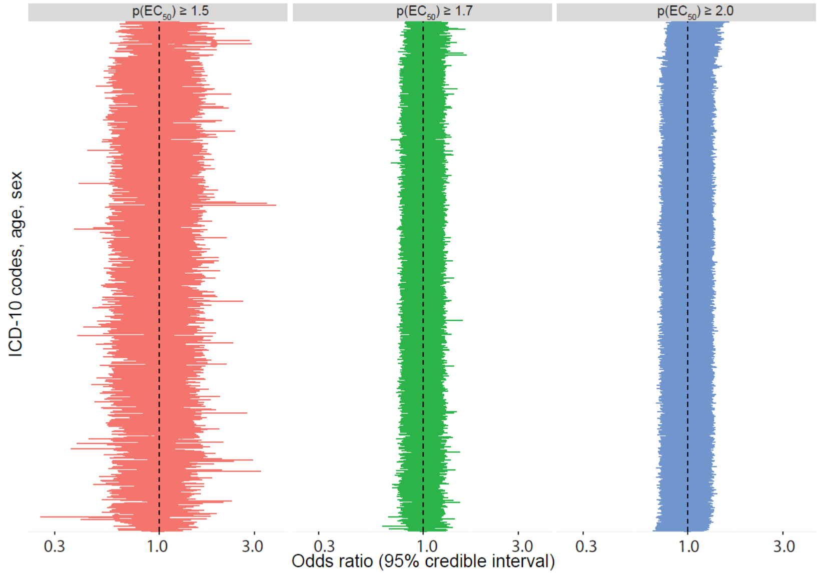

Appendix Fig S11. **Sensitivity analysis of different p(EC_{50}) cutoff values using multiple logisitc regression analysis**. Different p(EC_{50}) cutoff values that range from 1.5 (left), 1.7 (middle) to 2.0 (right). Y-axis: ICD-10 codes, age, and sex, which have been subjected to multiple logistic regression analysis using a Bayesian LASSO prior. X-axis: Odds ratio (OR) with 95% credible interval.

A

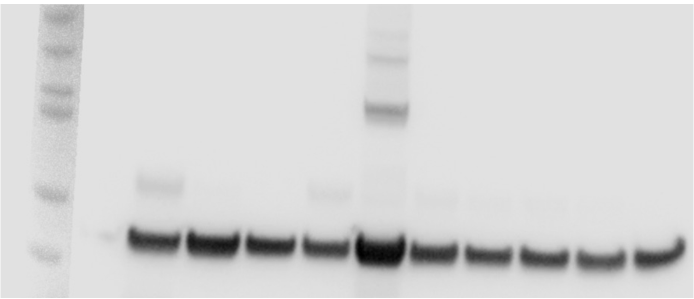

B

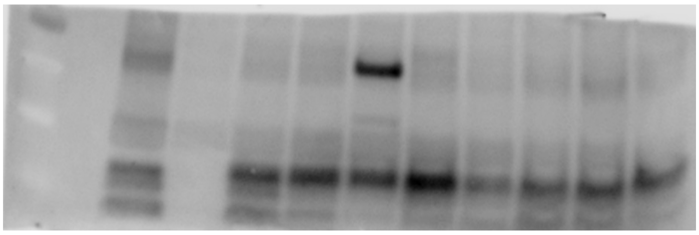

Appendix Fig S12. **Uncropped and unmodified western blot images of Fig 3**. **(A)** Uncropped image of western blot that was performed on spleen homogenate of AA^{+} mice and probed with anti-actin primary antibody. **(B)** Uncropped image of western blot that was performed on spleen homogenate of AA^{+} mice and probed with anti-SAA primary antibody for SAA detection. Of important note, the upper main band visible in lane 5 most likely represents an oligomeric SAA species.

A

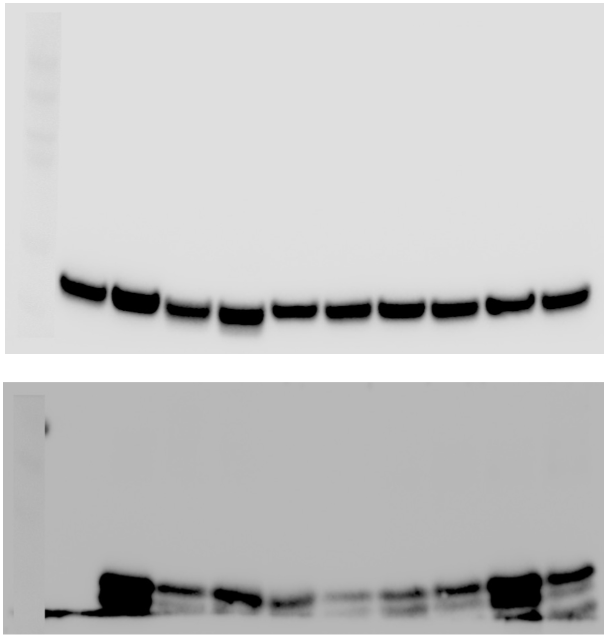

B

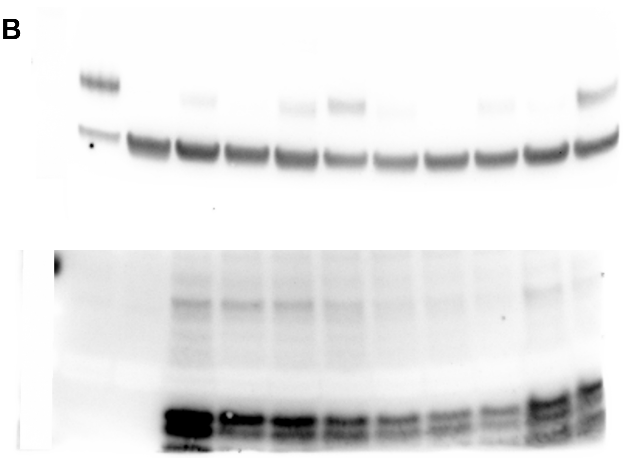

Appendix Fig S13. **Uncropped Western blot images of Fig 4**. **(A)** Uncropped image of Western blot that was performed on spleen homogenate of AA$^+$ mice and probed with anti-actin primary antibody (upper blot). Uncropped image of Western blot that was performed on spleen homogenate of AA$^+$ mice and probed with anti-SAA primary antibody for SAA detection (lower blot). **(B)** Second representative Western blot. Uncropped image of Western blot that was performed on spleen homogenate of AA$^+$ mice and probed with anti-actin primary antibody (upper blot). Uncropped image of Western blot that was performed on spleen homogenate of AA$^+$ mice and probed with anti-SAA primary antibody for SAA detection (lower blot). Of note: Small part of the membrane (last lane in the lower blot) was cut during processing.

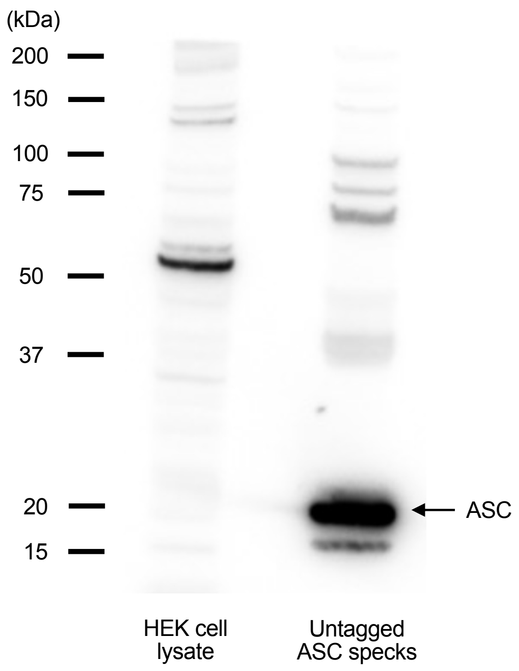

Appendix Fig S14. Purified ASC speck assessment by western blot. Uncropped image of western blot that was performed on HEK cell lysate (control, left lane) and purified untagged ASC specks (right lane) and probed with anti-ASC primary antibody for ASC detection and assessment. Different bands and respective sized in the right lane represent ASC oligomers.

Appendix Table S1. *Accelerated SAA fibrillation in the presence of ASC specks.*

ASC specks (nr #) / Time ($t_{1/2}$)	0	1 x 10^5	2 x 10^5	5 x 10^5
Mean ± SD (hours)	98.83 ± 8.16	82.12 ± 8.73	79.22 ± 7.18	66.30 ± 4.10

Appendix Table S2. *Temporal SAA serum concentrations upon silver nitrate injection or AA induction.*

	Pycard$^{+/+}$		*Pycard*$^{-/-}$	
Group / Time (hrs)	$AgNO_3$ Mean ± SD (µg/ml)	$AgNO_3$+AEF Mean ± SD (µg/ml)	$AgNO_3$ Mean ± SD (µg/ml)	$AgNO_3$+AEF Mean ± SD (µg/ml)
Baseline	6.08 ± 6.44	1.49 ± 1.22	1.39 ± 0.99	1.27 ± 1.06
9h	84.44 ± 15.22	63.41 ± 36.12	49.08 ± 38.48	80.98 ± 49.2
24h	217.1 ± 124.5	125.4 ± 25.04	188.5 ± 120	231.9 ± 97.13
48h	118 ± 60.21	95.2 ± 40.01	106.5 ± 65.24	149 ± 34.62
72h	19.22 ± 10.65	16 ± 5.94	16.16 ± 7.7	26.69 ± 10.79
96h	3.28 ± 0.47	3.48 ± 1.55	4.38 ± 0.79	7.04 ± 5.77

Appendix Table S3. *HS-310-stained AA amyloid.*

	Pycard$^{+/+}$		*Pycard*$^{-/-}$	
Treatment / Group	$AgNO_3$ Mean ± SD (HS-310 area, %)	$AgNO_3$+AEF Mean ± SD (HS-310 area, %)	$AgNO_3$ Mean ± SD (HS-310 area, %)	$AgNO_3$+AEF Mean ± SD (HS-310 area, %)
16- day	-	6.13 ± 4.48	-	3.11 ± 2.46
30-day	-	17.63 ± 8.38	-	8.93 ± 6.5

Appendix Table S4. *Phagocytosis of Pycard$^{+/+}$ and Pycard$^{-/-}$ BMDMs.*

Genotype / Condition BMDMs	*Pycard*$^{+/+}$ Mean ± SD OD at 405 nm	*Pycard*$^{-/-}$ Mean ± SD OD at 405 nm	*p*-value	Sign.
Unstimulated	1.58 ± 0.03	1.53 ± 0.09	0.393	ns
Unstimulated + Cytochalasin D	0.32 ± 0.03	0.35 ± 0.03	0.077	ns
mSAA stimulated	1.72 ± 0.05	1.55 ± 0.11	0.002	sign.
mSAA stimulated + Cytochalasin D	0.34 ± 0.05	0.34 ± 0.01	0.630	ns

Appendix Table S5. *Genotype and sex of experimental animals.*

Group / Gender	AEF+$AgNO_3$ 16- and 30-day group	$AgNO_3$ only 16- and 30-day group	PBS only	AEF only	Total (n =)
Pycard$^{-/-}$ females (n =)	10	4	3	1	18
Pycard$^{-/-}$ males (n =)	14	6	0	3	23
Pycard$^{+/+}$ females (n =)	10	4	0	0	14
Pycard$^{+/+}$ males (n =)	12	6	3	3	24
Total (n =)	46	20	6	7	79

Appendix Table S6. *Genotype and age of experimental animals.*

Genotype / Treatment	*Pycard*$^{+/+}$	*Pycard*$^{-/-}$	*p*-value	Sign.
**PBS-only** (mean days ± SD)	77.67 ± 1.16	58.33 ± 19.63	0.164	ns
**AEF-only** (mean days ± SD)	80.33 ± 1.16	59.75 ± 15.17	0.071	ns
**$AgNO_3$-only** (mean days ± SD)	100.0 ± 20.07	94.8 ± 21.14	0.580	ns
**$AgNO_3$ + AEF** (mean days ± SD)	81.68 ± 12.11	88.96 ± 16.53	0.101	ns
**Total** (mean days ± SD)	86.08 ± 15.93	85.20 ± 21.18	0.837	ns

Appendix **Table S7.** Sex of experimental immunotherapy animals

Treatment / Gender	AEF+$AgNO_3$ (AA induction)	AA + anti-ASC abs	AA + isotype abs	Total (n =)
Pycard$^{+/+}$ females (n =)	4	3	4	11
Pycard$^{+/+}$ males (n =)	1	2	1	4
Total (n =)	5	5	5	15

Appendix Table S8. *Age of experimental immunotherapy animals.*

Genotype / Treatment	*Pycard*$^{+/+}$	*p*-value	Sign.
**1.) AA induction only** (mean weeks ± SD)	19.8 ± 5.59	1 vs 2: 0.959	ns
**2.) AA + anti-ASC abs** (mean weeks ± SD)	18.8 ± 4.76	2 vs 3: 0.974	ns
**3.) AA + isotype abs** (mean weeks ± SD)	19.6 ± 6.73	3 vs 1: 0.998	ns
**Total** (mean weeks ± SD)	19.4 ± 5.34	0.959	ns

***Appendix* Table S9.** Western blot antibodies.

Antibody	Clone	Host / Conjugate	Source	Dilution (µl)
SAA recombinant rabbit monoclonal	D9H4L41	Rabbit	Invitrogen, #700830	1:200
Goat anti-rabbit IgG (H+L)	-	Goat/ HRP	Jackson Immuno, #111-035-045	1:5000
Anti-Actin antibody monoclonal	C4	Mouse	Merck, #MAB1501R	1:8000
Goat anti-mouse IgG (H+L)	-	Goat/ HRP	Jackson Immuno, #115-035-003	1:8000

***Appendix* Table S10.** Flow cytometry antibodies.

Surface antigen	Fluorophore	Source	Dilution (µl)
Anti-mouse B220	Brilliant Violet 785	Biolegend, #103245	1:400
Anti-mouse CD11b	Brilliant Violet 650	Biolegend, #101239	1:400
Anti-mouse CD45.2	Pacific Blue	Biolegend, #109820	1:100
Anti-mouse CD3	Per/CPCy5.5	Biolegend, #100218	1:100
Anti-mouse Gr-1	PE/Cy5	eBioscience™, #15-5931-82	1:400
Anti-mouse CD206	PE	Biolegend, #141705	1:100
Anti-mouse F4/80	APC-eFluor 780	eBioscience™, #47-4801-82	1:200
Anti-mouse MHCII	Alexa Fluor® 700	Biolegend, #107621	1:800
Anti-mouse CD11c	APC	eBioscience™, #17-0114-82	1:400

***Appendix* Table S11.** Antigens used for the high-throughput antibody profiling.

Antigen	Application	Coating concentration	Source	Product #
ASC-C-his	ELISA	1 µg/ml	Matthias Geyer, ISB, University of Bonn (Venegas *et al*, 2017)	
human $recPrP_{23\text{-}230}$	ELISA	1 µg/ml	In-house (Senatore *et al*, 2020)	
Tau441	ELISA	2 µg/ml	In-house	
SARS-CoV-2 Spike ECD	ELISA	1 µg/ml	(Emmenegger *et al*, 2021)	
nAra h 2	ELISA	1 µg/ml	Indoor Biotechnologies	NA-AH2-1
ASC-PYD	ELISA	1 µg/ml	Mabylon AG, Schlieren	
ASC-CARD	ELISA	1 µg/ml	Mabylon AG, Schlieren	
LAG3	ELISA	1 µg/ml	AcroBiosystems	LA3-H5222
TIM3	ELISA	1 µg/ml	AcroBiosystems	TM3-H5229

***Appendix* Table S12.** Antibodies used for the high-throughput antibody profiling.

Species	Target	Dilution/Concentration range	Brand name	Product #
(HRP) Goat	anti-human IgG	1:4000	Jackson	109-035-098
(HRP) Goat	anti-mouse IgG	1:2000	Jackson	115-035-003
(HRP) Goat	anti-rabbit IgG	1:2000	Jackson	111-035-045
Mouse	anti-ASC	1 µg/ml - 0.06 ng/ml	Santa Cruz	sc-514414
Human	anti-human PrP	1 µg/ml - 0.06 ng/ml	In-house	
Mouse	anti-Tau441	1 µg/ml - 0.06 ng/ml	Sigma-Aldrich	05-804
Mouse	anti-his	1 µg/ml - 1,38 ng/ml	Invitrogen	37-2900
Rabbit	anti-ASC	1 µg/ml - 1,38 ng/ml	Mabylon AG, Schlieren	MY6745

References

Emmenegger M, Kumar SS, Emmenegger V, Malinauskas T, Buettner T, Rose L, Schierack P, Sprinzl MF, Sommer CJ, Lackner KJ *et al* (2021) Anti-prothrombin autoantibodies enriched after infection with SARS-CoV-2 and influenced by strength of antibody response against SARS-CoV-2 proteins. *PLoS Pathog* 17: e1010118

Jagusiak A, Rybarska J, Konieczny L, Piekarska B, Stopa B, Chłopaś K, Zemanem G, Roterman I (2019) Amyloids, Congo red and the apple-green effect. *Acta Biochim Pol* 66: 39-46

Senatore A, Frontzek K, Emmenegger M, Chincisan A, Losa M, Reimann R, Horny G, Guo J, Fels S, Sorce S *et al* (2020) Protective anti-prion antibodies in human immunoglobulin repertoires. *EMBO Mol Med* 12: e12739

Venegas C, Kumar S, Franklin BS, Dierkes T, Brinkschulte R, Tejera D, Vieira-Saecker A, Schwartz S, Santarelli F, Kummer MP *et al* (2017) Microglia-derived ASC specks cross-seed amyloid-β in Alzheimer's disease. *Nature* 552: 355-361
