# Supplementary figures and images for: The ASC inflammasome adapter governs SAA-derived protein aggregation in inflammatory amyloidosis
Source: EMBO Mol Med. 2024 Jul 30;16(9):2024–42. doi: 10.1038/s44321-024-00107-0 (PMC11393341; doi:10.1038/s44321-024-00107-0)

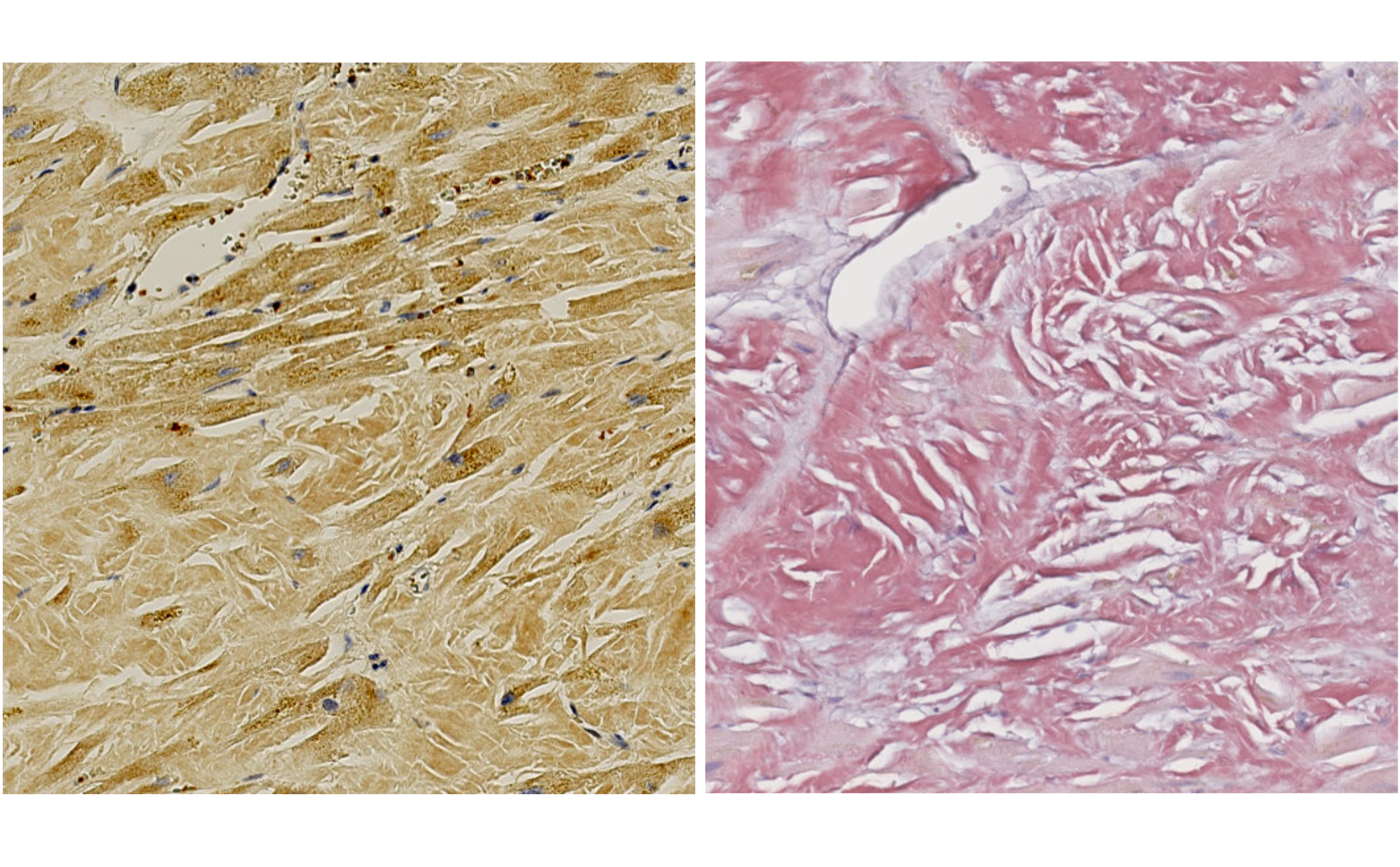

Supplement: Supplementary file 3 — Source data Fig. 1 [file 44321_2024_107_MOESM3_ESM.zip › Source data_Figure 1/Fig 1B.tiff]

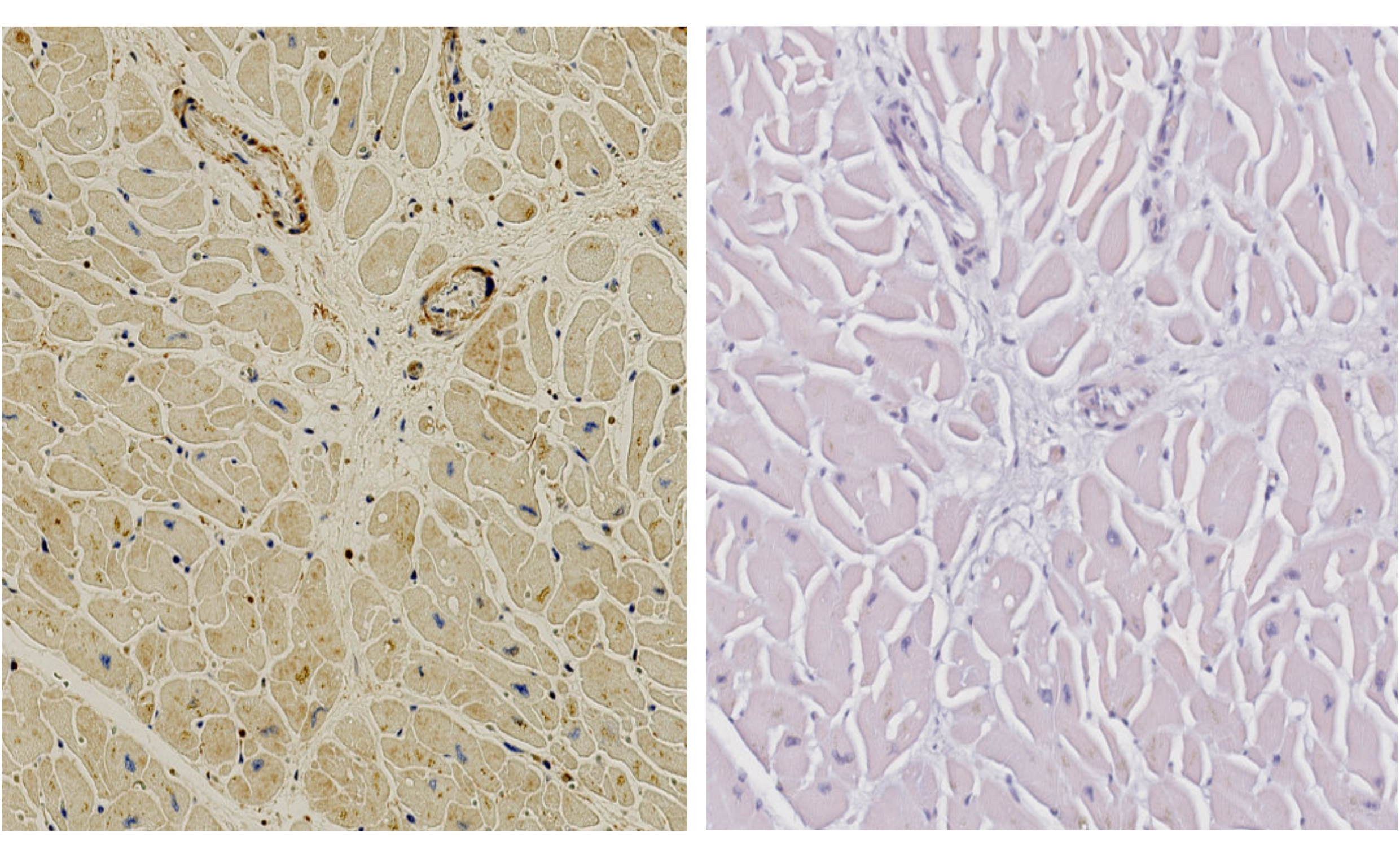

Supplement: Supplementary file 3 — Source data Fig. 1 [file 44321_2024_107_MOESM3_ESM.zip › Source data_Figure 1/Fig 1A.tiff]

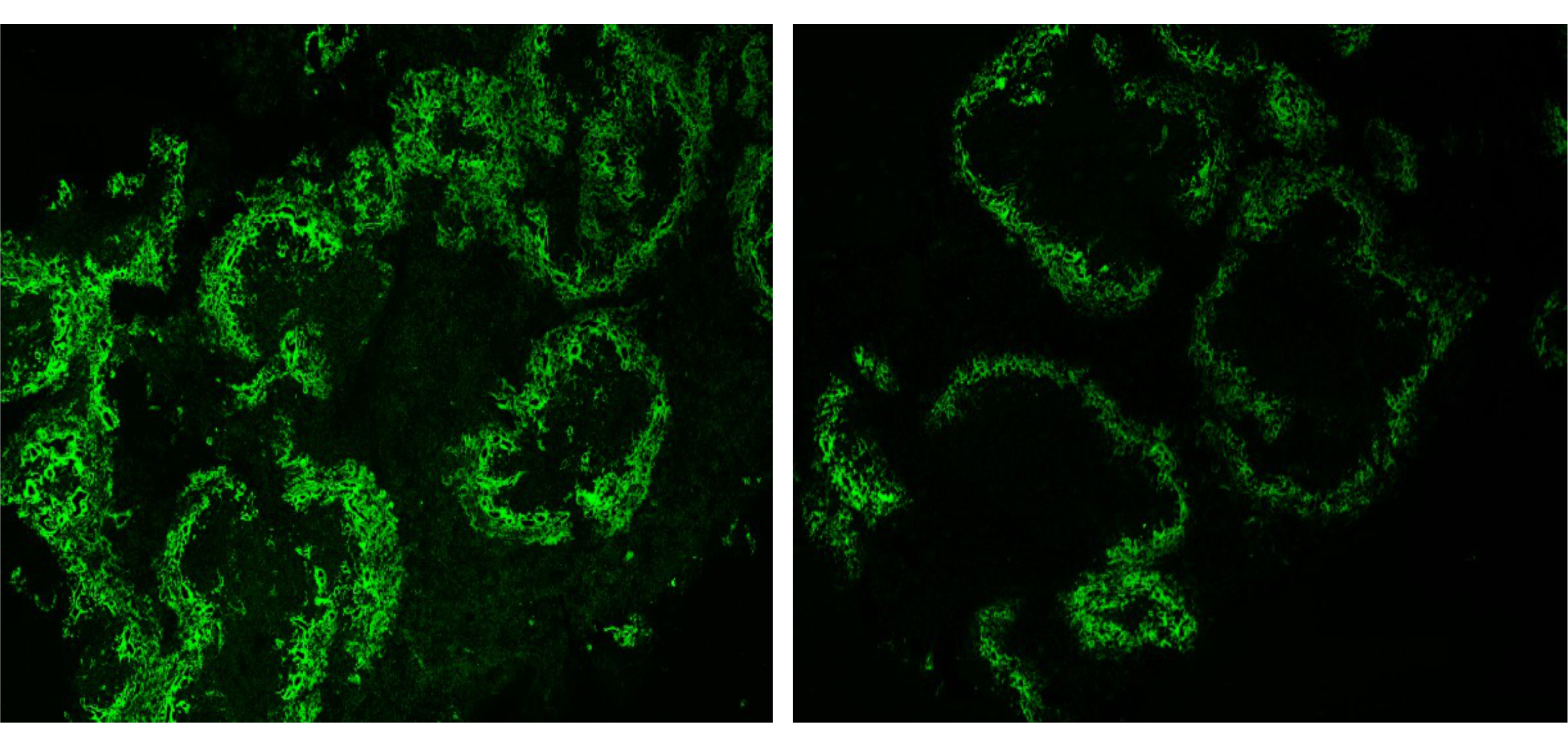

Supplement: Supplementary file 5 — Source data Fig. 3 [file 44321_2024_107_MOESM5_ESM.zip › Source data_Figure 3/Fig 3C.tiff]

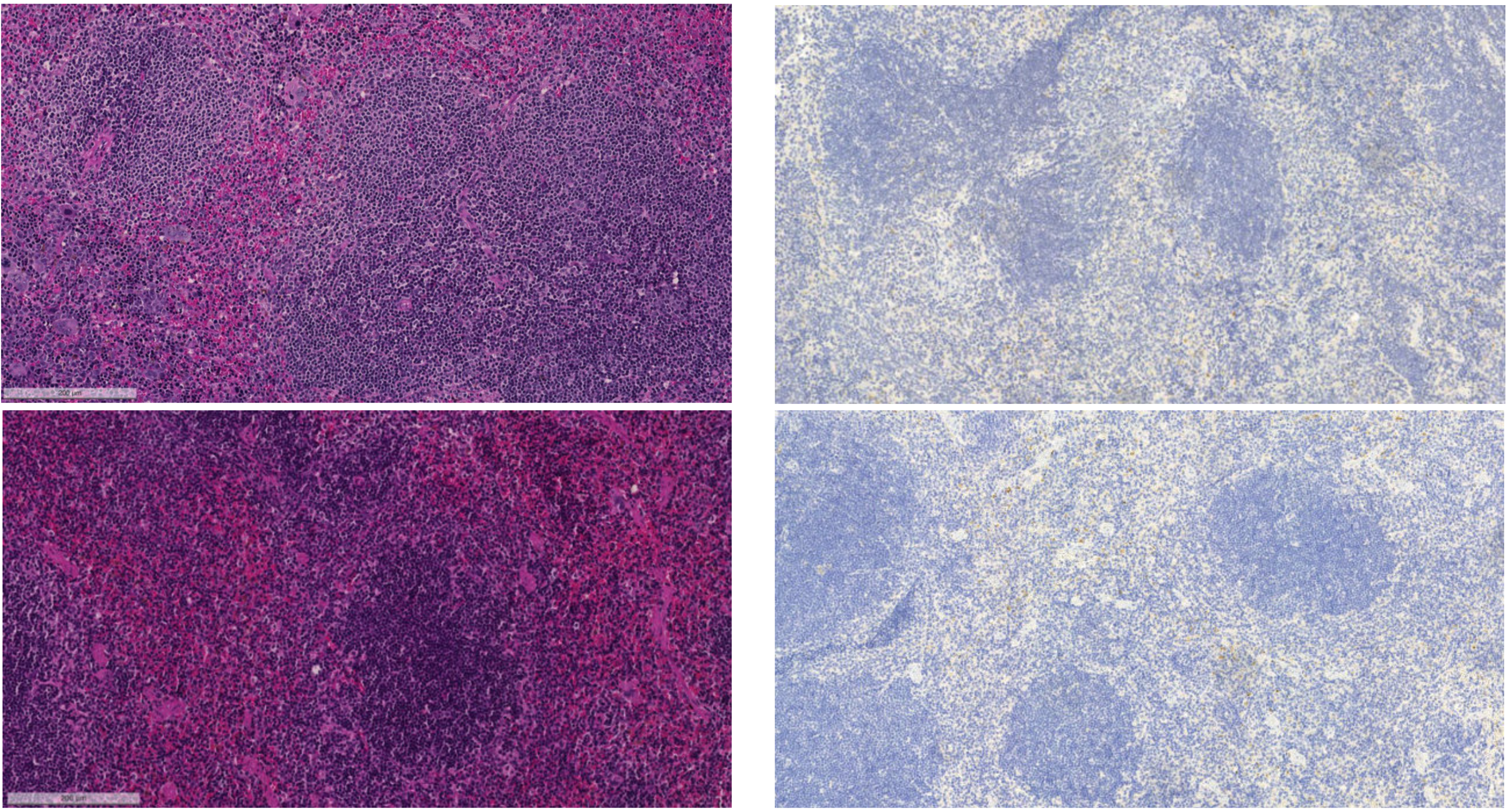

Supplement: Supplementary file 5 — Source data Fig. 3 [file 44321_2024_107_MOESM5_ESM.zip › Source data_Figure 3/Fig 3G.tiff]

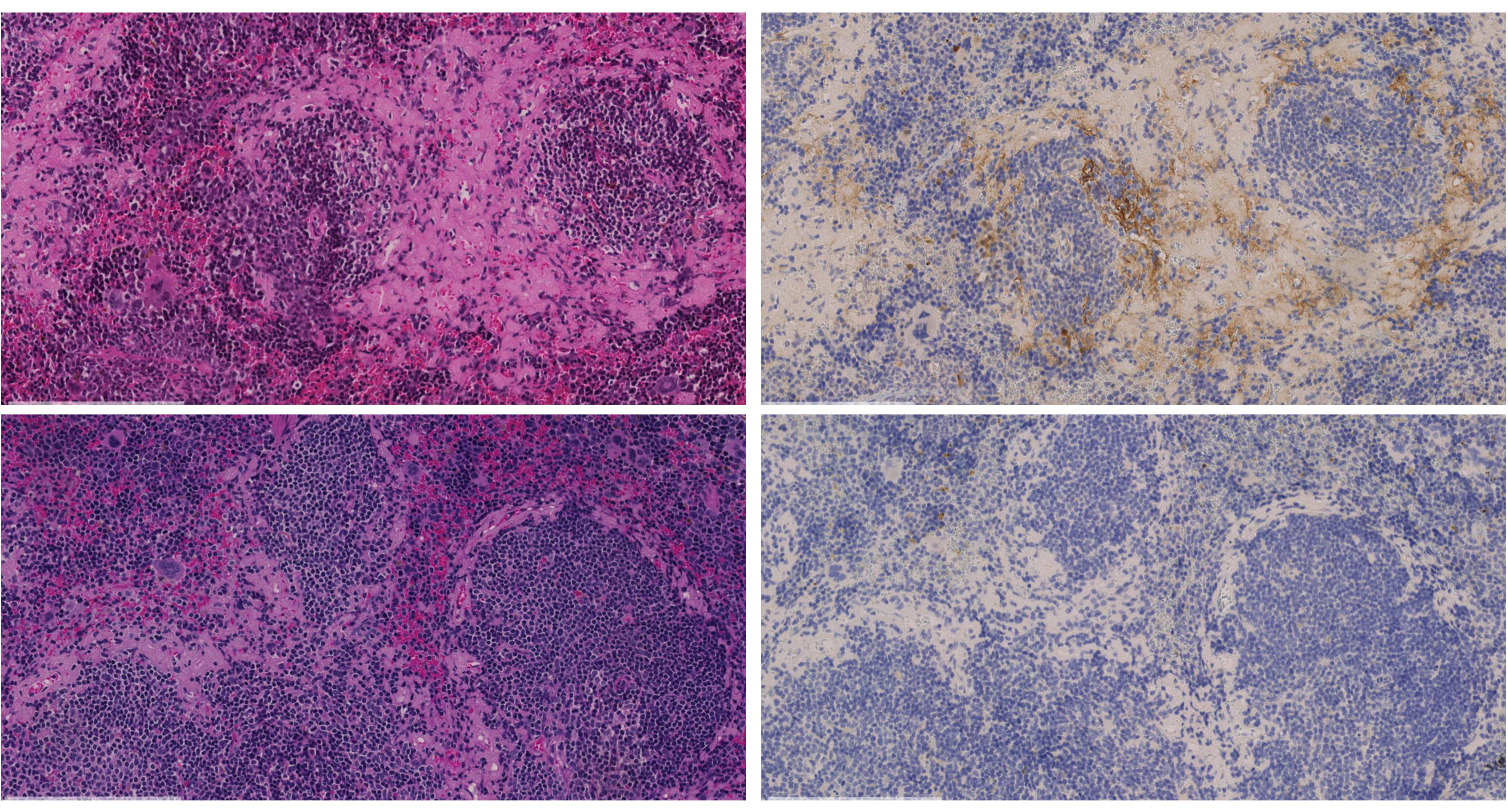

Supplement: Supplementary file 5 — Source data Fig. 3 [file 44321_2024_107_MOESM5_ESM.zip › Source data_Figure 3/Fig 3F.tiff]

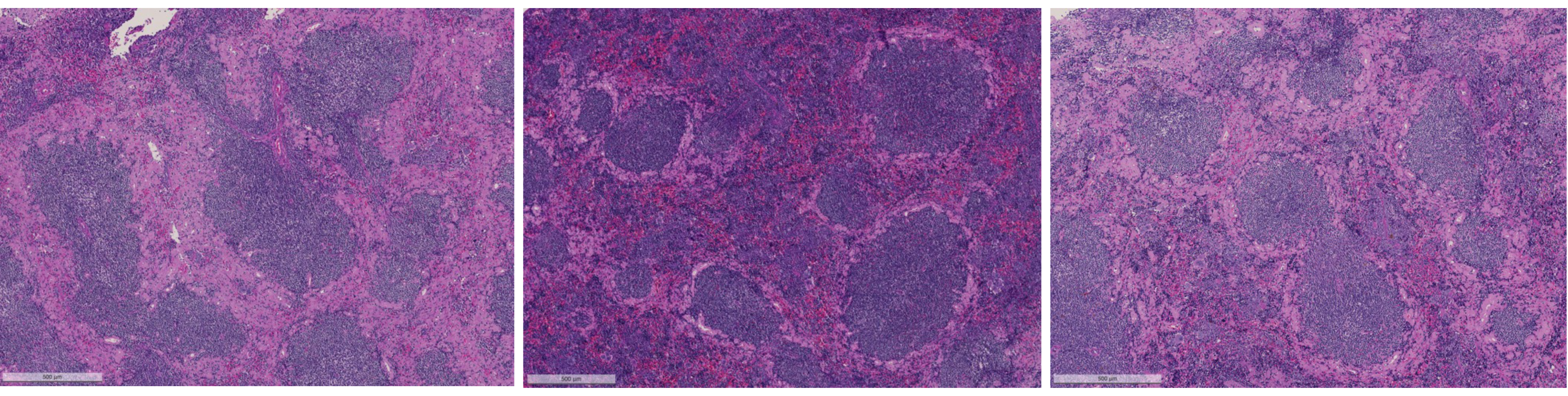

Supplement: Supplementary file 6 — Source data Fig. 4 [file 44321_2024_107_MOESM6_ESM.zip › Source data_Figure 4/Fig 4D.tiff]

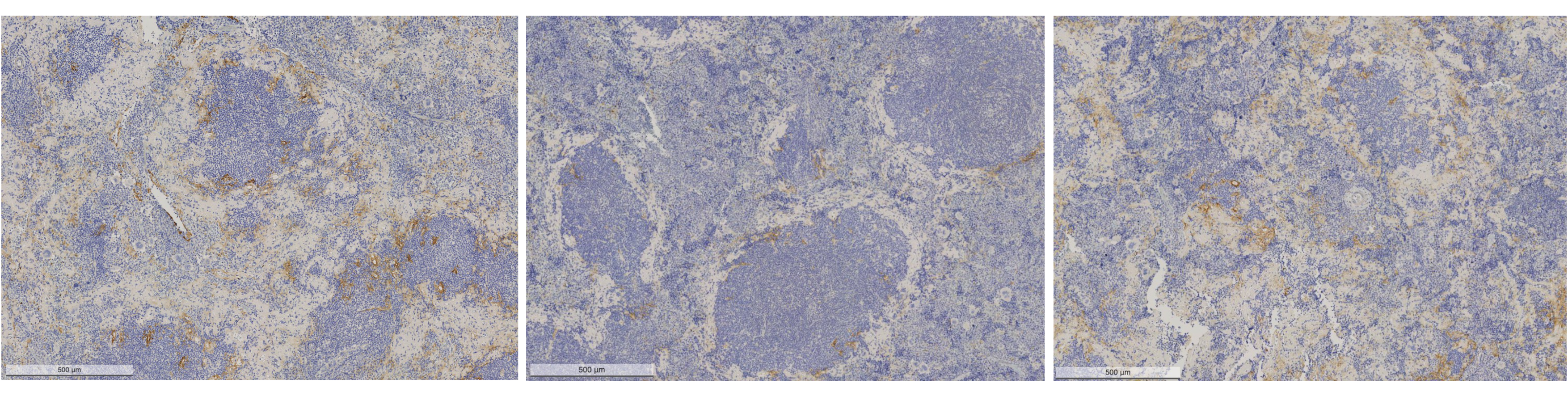

Supplement: Supplementary file 6 — Source data Fig. 4 [file 44321_2024_107_MOESM6_ESM.zip › Source data_Figure 4/Fig 4E.tiff]

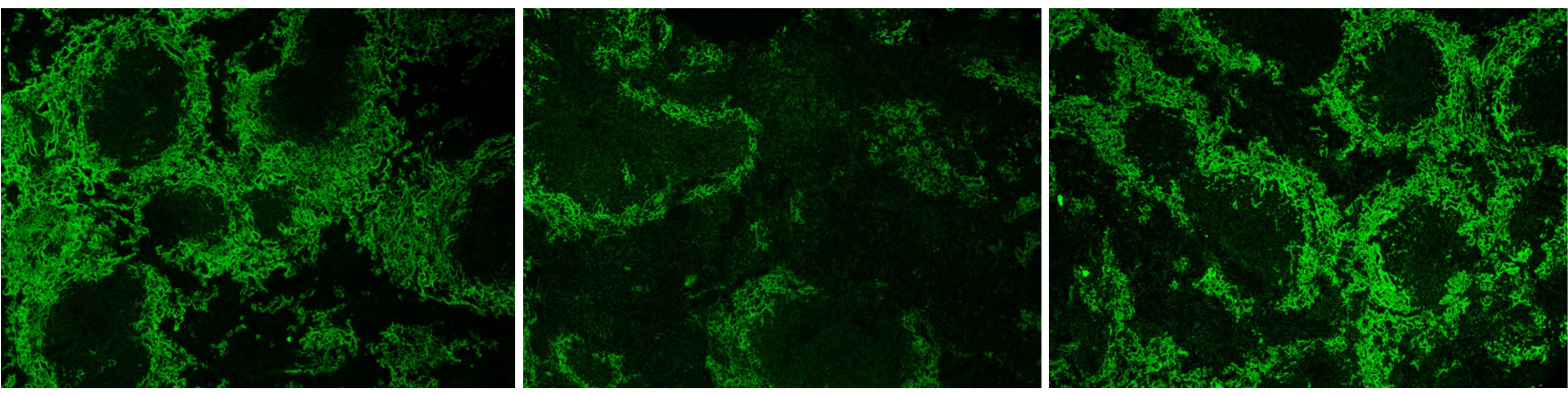

Supplement: Supplementary file 6 — Source data Fig. 4 [file 44321_2024_107_MOESM6_ESM.zip › Source data_Figure 4/Fig 4G.tiff]
